# Enhanced Cell Adhesion and Alignment on Micro-Wavy Patterned Surfaces

**DOI:** 10.1371/journal.pone.0104502

**Published:** 2014-08-08

**Authors:** Jia Hu, Camille Hardy, Chi-Mon Chen, Shu Yang, Arkady S. Voloshin, Yaling Liu

**Affiliations:** 1 Department of Mechanical Engineering and Mechanics, Lehigh University, Bethlehem, Pennsylvania, United States of America; 2 Bioengineering Program, Lehigh University, Bethlehem, Pennsylvania, United States of America; 3 Department of Material Science, University of Pennsylvania, Philadelphia, Pennsylvania, United States of America; University of California, San Diego, United States of America

## Abstract

Various micropatterns have been fabricated and used to regulate cell adhesion, morphology and function. Micropatterns created by standard photolithography process are usually rectangular channels with sharp corners (microgrooves) which provide limited control over cells and are not favorable for cell-cell interaction and communication. This paper proposes a new micropattern with smooth wavy surfaces (micro-waves) to control the position and orientation of cells. To characterize cell growth and responses on the micro-patterned substrates, bovine aortic endothelial cells were seeded onto surfaces with micro-grooves and micro-waves for 24 h. As a result, the cells on the micro-wavy pattern appeared to have a lower death rate and better alignment compared to those on the micro-grooved pattern. In addition, flow-induced shear stress was applied to examine the adhesion strength of cells on the micro-wavy pattern. Results showed that cells adhered to the wavy surface displayed both improved alignment and adhesion strength compared to those on the flat surface. The combination of increased alignment, lower death rate and enhanced adhesion strength of cells on the micro-wavy patterns will offer advantages in potential applications for cell phenotype, proliferation and tissue engineering.

## Introduction

In recent years, significant efforts have been made to develop various nano/micro-patterned substrates for application in biomedical systems [1], [2]. A range of factors affect the behaviors of cells on a substrate, including surface chemistry, feature geometry and elastic modulus [3]. The behavior of embryonic cells was first investigated by Harrison [4]. Cells movement and orientation behavior was named ‘contact guidance’ by Weiss [5]. Curtis and Varde investigated the cell behavior and morphology on silica substrates of various topology [6]. The topographical control of cell behavior was widely examined by researchers during the past five decades [7], [8], [9]. The ability to control the position of cells in an organized pattern on a substrate has become increasingly important for the development of cellular biosensor technology and tissue engineering applications [10], [11], [12].

In tissue engineering, cells are often organized in a particular pattern, such as those in a neural network [13] and in a liver system [14]. In recent years, there have been developments of growing a biomimetic tissue in microfluidic devices, namely “tissue-on-a-chip”. Various techniques are applied to place cells into a pre-designed functional pattern, such as physical and chemical cell trapping [15], [16], substrate topography [17], fluid shear [18], and compression [19]. Liu *et al.* fabricated a chip that can organize several thousands of cells into an artificial liver [20]. Electrophoresis is used to arrange specialized liver cells (hepatocytes) into chains radiating from a central point [20].

With the advent of nanofabrication techniques, the effects of nano-scale grooved patterns on cell spreading, migration, morphology, and orientation has been studied [21], [22], [23]. Recent studies have shown that cell orientation and differentiation can be influenced by a nanostructured surface [24]. Peterbauer et al. show that cell orientation follows the orientation of initial protrusions from the cell when seeded onto a nano-grooved surface [24]. Meanwhile, Wang et al. observed that submicron grooved pattern inhibited proliferation of skeletal muscle cells, yet enhanced its differentiation capabilities [25]. Their results also supported others' studies that cells align along the grooves of the substrates. Nevertheless, nanogrooves cannot provide a good control of cell location since the groove dimensions are much smaller than the cell size. To precisely control the cell location, it is desirable to use a micro-pattern with size similar to the cell.

Researchers have studied the effects of micro-patterned geometry on cell spreading, migration and alignment on poly(dimethylsiloxane) (PDMS) [26], [27], [28], [29]. For example, Fu *et al.* demonstrate that the spacing and height of microgrooved PDMS can influence spreading and adhesion of human osteosarcoma (HOS) cells [30]. Culturing HOS cells on microgrooves with spacing ranging from 5 µm to 120 µm shows that cells align well along the directions of microgrooves when the groove spacing is comparable to the spread cell size [30].

Micropatterns created by standard photolithography process are usually rectangular channels with sharp corners (microgrooves). In most studies reported so far, cells are placed on these microgrooves. While these grooves provide strong geometry constrain on cells, these individually separated microgrooves cannot provide contact interfaces between cells, which are important for cell-cell interaction study. Additionally, cells on the microgrooved substrate exhibited significantly lower proliferation rates compared to those on the flat surface [31].

So far, there has not been a technique that can provide good control of both cell position and orientation while allowing for cell-cell interaction. In this work, we fabricated micropatterns with sinusoidal waves and investigated adhesion and alignment of BAOECs on the curved surface. While most current studies focus on a flat surface, cells are usually interacting on a non-flat surface. For example, in capillary vessel with a diameter of 5–10 µm [32], the endothelium cells are found covering a highly curved surface. It raises a fundamental and interesting question how surface curvature cue may determine cell behaviors. A lot of artificial implants also have micropatterns on the surface. For example, human umbilical vein endothelial cells were seeded onto 4 mm I.D. expanded poly(tetrafluoroethylene) (e-PTFE) grafts [33]. Understanding how cells interact with a curved surface is also essential for enhancing cell seeding and growth on these artificial materials.

This paper presents the results of an experimental study of the spreading and adhesion of cells on wavy surfaces in comparison to grooved and flat surfaces. We first fabricated smooth micro-wavy patterns with 20 µm wavelength and 6.6 µm height, and micro-grooved patterns with 20 µm spacing and 5 µm height. Then, cell spreading, alignment and death were investigated through microscopy after a 24 h incubation period. Finally, we packed a microfluidic device and place the cells in the device, then apply shear flow to the device to examine the adhesion strength of BAOECs on the micro-wavy pattern compared to that on the flat and grooved surface. The implications of the results are then discussed for the application as a tissue scaffold pattern.

## Materials and Methods

**Figure 1:**
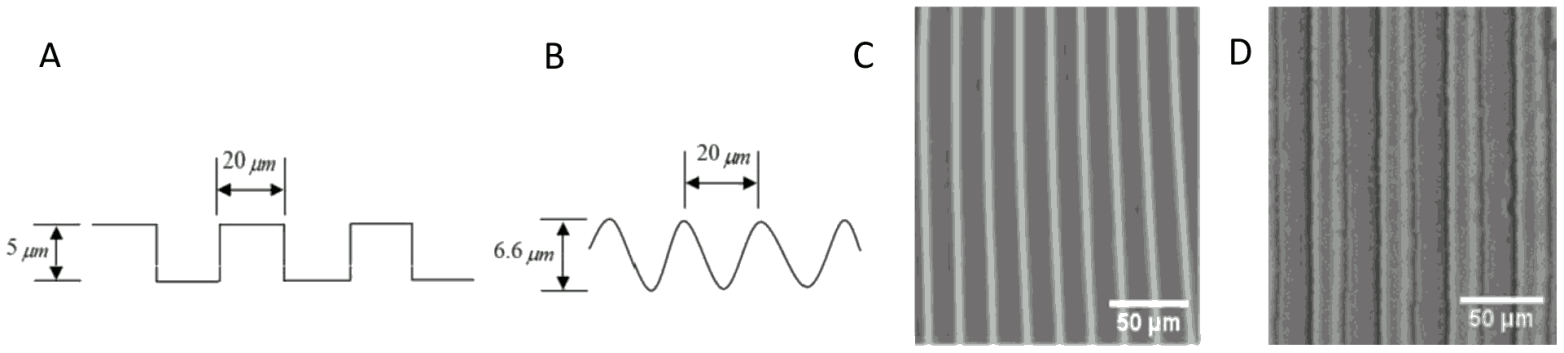
Geometry of micropatterns. (A) Micro-groove cross-sectional figure; (B) Micro-wave cross-sectional figure; (C) 1D 20 µm wavelength wavy pattern; (D) 1D 20 µm groove length groove pattern.

To characterize cell growth and responses on a micro-patterned substrate, Bovine Aortic Endothelial Cells (BAOECs) were seeded onto micro-groove (Fig. 1A and 1D) and micro-wavy (Fig. 1B and 1C) substrates.

### Micro-patterned PDMS substrates fabrication

**Figure 2:**
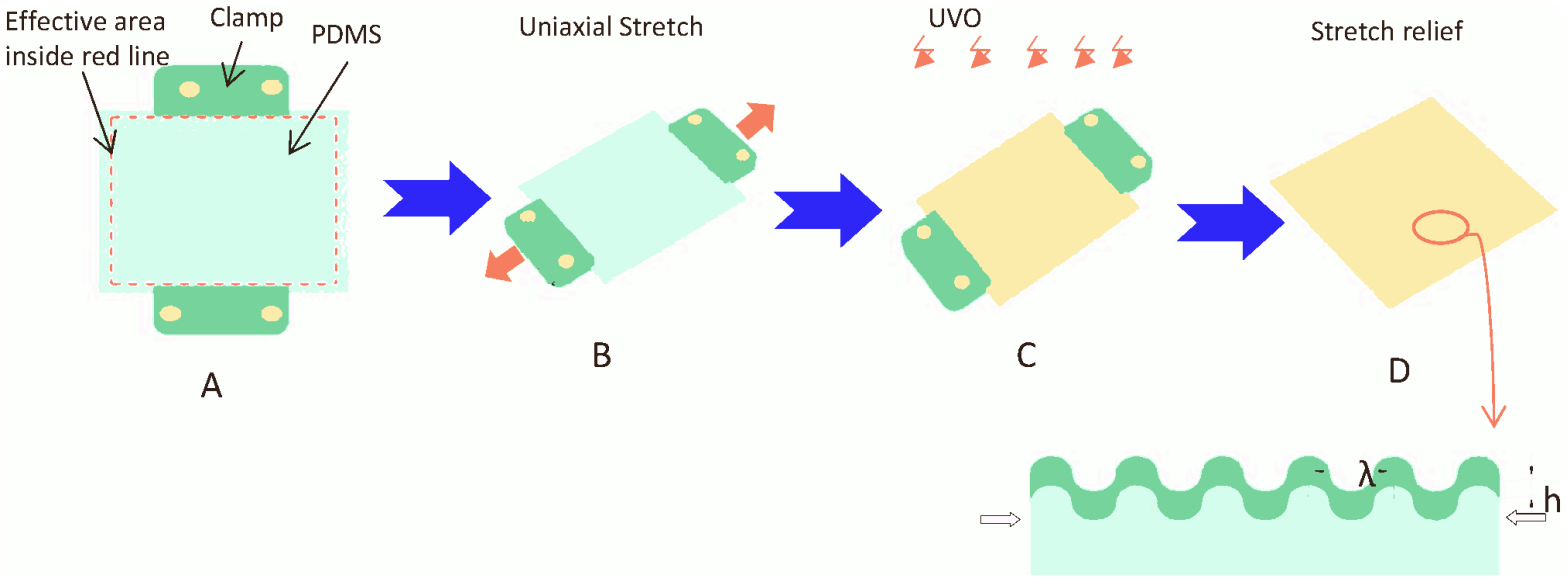
Illustration of the fabrication process of microwavy patterns. (A–D) Uniaxial stretching of PDMS films at various mechanical stretch settings to generate microwavy patterns.

Microgrooved surfaces used in cell capture or particle isolation can be fabricated through standard photolithography, followed by PDMS molding techniques [24]. However, these surfaces typically have sharp corners, which are not favorable for cell seeding, spreading, and adhesion [28], [30]. To fabricate a smooth microwavy surface, we used bucking of oxide/PDMS bilayer, as shown in Fig. 2.

Basically, a flat 0.5 mm thick PDMS sheet (40 mm×15 mm) was prepared from Sylgard 184 (Dow Corning, Midland, MI, USA) in a 10∶1 weight ratio of silicone elastomer base to the curing agent. After curing at 65°C for 4 h, the crosslinked PDMS sheet was then uniaxially stretched by a custom-designed stretching device to 50.0% strain and exposed to a UVO cleaner (model 144AX, Jelight Company, Inc.) or O_2_ plasma (Enercon Industries Corp., Menomonee Falls, WI, USA) for 1 hour to generate a thin silicate layer [34], [35]. After releasing the substrate from the stretcher, the difference in elastic modulus causes the oxide/PDMS bilayer to buckle and form wrinkled surface. The wavelength (λ) and amplitude (A) of the wrinkles can be tuned by varying the elastic modulus of the PDMS substrate and the thickness of the top silicate layer [35], [36], [37], [38]. The micro-wavy pattern used in this study was created with a wavelength of 20 µm and a height of 6.6 µm.

We also fabricated micro-grooved molds on silicon wafers through photolithography with geometries shown in Fig. 1 D. PDMS was then poured into the molds. After degassing in vacuum chamber for 30 min, the samples were cured in an oven at 65°C for 4 hours. The cured PDMS layers were peeled off. The micro-grooved patterns were created with 20 µm spacing and 5 µm height.

### Microfluidic device fabrication

The microfluidic shear devices were assembled by covering the fabricated micropatterned wavy surface with a PDMS transparent cover pattern. A desktop digital craft cutter (Silhouette America, Inc., UT, USA) was used to make the cover pattern [39]. By using the interactive computer software, 120 µm thick adhesive gold foil (Silhouette America Inc., UT, USA) was printed to form the design of the structure. This foil was stuck onto the glass as a cover pattern mold. PDMS was poured onto the mold. The cured PDMS layers were peeled off after 4 h at 65°C. The cover pattern was a rectangle 36 mm long and 6 mm wide. Two 2 mm diameter holes were punched on the two sides of cover pattern as the inlet and outlet. The PDMS substrate and cover pattern were both rinsed in 70% ethanol, plasma treated for 90 seconds, and then bound together to assemble microfluidic channel devices.

### Cell culture on micro-patterned substrates

All reagents and chemicals for cell culture and detachment were purchased from Sigma-Aldrich USA. Our endothelial cells were obtained from Cell Applications (San Diego, CA). BAOECs were maintained in 25 cm^2^ cell culture flasks that were kept at 37°C in Dulbecco's Modified Eagle's medium (DMEM) containing 10% heat inactivated Fetal Bovine Serum (FBS) and 1% Penicillin/Streptomycin in a 100% humidity atmosphere with 5% CO_2_. BAOECs between passages 10–15 were used in all reported experiments.

Prior to static cell culture experiment, the PDMS substrates were sterilized by rinsing with 70% ethanol (2 h), followed by phosphate buffered saline (PBS) (5 min) and autoclaved for 1 h. Gelatin was used in this study to increase cell attachment to substrate. It was diluted to desired concentration (0.2%) with TE buffer. The micropattern surfaces were rinsed with 0.2% gelatin for 2 h in the incubator to allow sufficient gelatin adsorption onto the underlying PDMS surface. After rinsing with 1 ml PBS, micropatterns were rinsed and maintained in supplemented medium until cell seeding.

Determined by a hemocytometer count, approximately 2.5×10^5^ cells/mL concentrated cells were seeded by standard cell culture protocol onto the micro-patterned PDMS substrates. After 24 h and 48 h incubation, cell spreading and orientation was then imaged with a Nikon phase-contrast microscope.

### Cell death

Cell death was determined by staining with ethidium homodimer-1 (EthD-1) (Sigma Aldrich, USA), a fluorescent nuclear stain that penetrates dead cells and increases intensity after binding to DNA. Staining was done according to the standard procedures. Staining cells on different patterns were counted in 3 randomly selected microscopic fields (at least 500 cells) and the % positive cells were calculated relative to the total number of cells on the pattern. Cell death rate data are expressed as the mean % positive cells ± SEM.

### Cell adhesion strength assays

**Figure 3:**
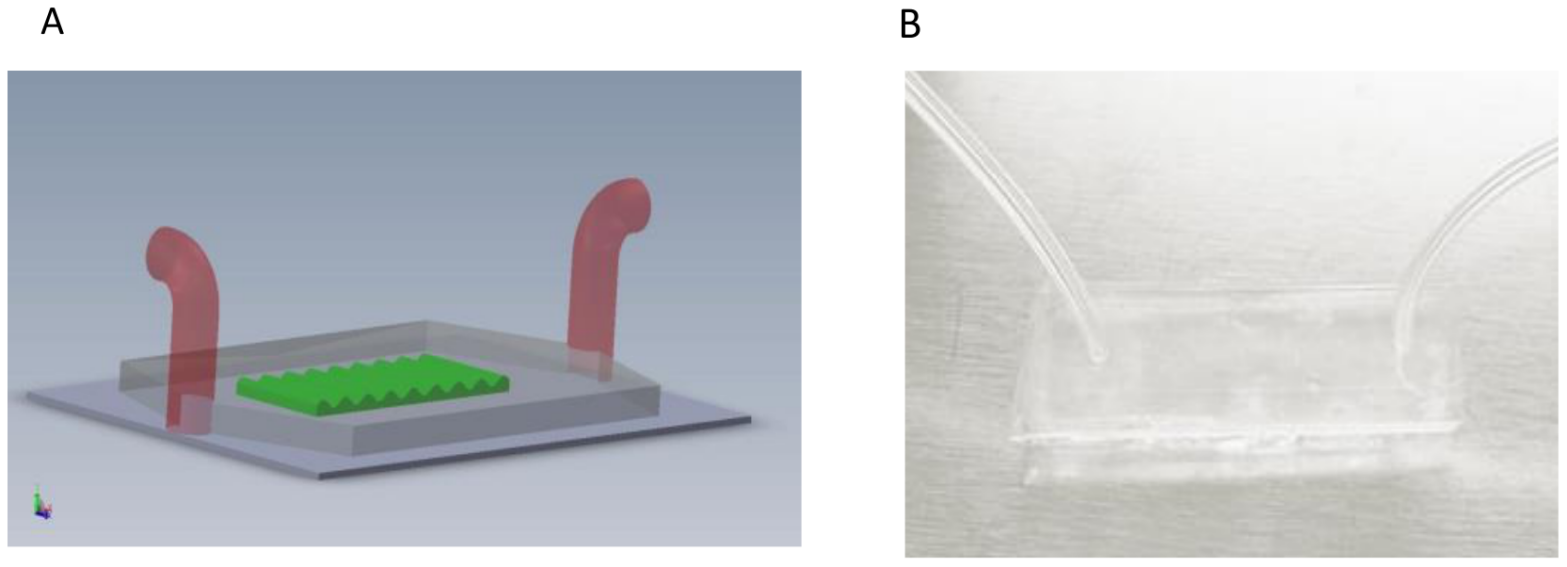
Microfluidic based testing device. (A) Sketch of the microfluidic device; (B) Image of the microfluidic device.

Besides static seeding, cell response to external stimuli such as shear flow was also studied via a microfluidic based testing platform. A typical integrated microfluidic testing device is illustrated in Fig. 3 A–B. The fabricated micropatterned surface was covered with a PDMS transparent cover pattern via O_2_ plasma bonding [40], [41]. As a comparison, a flat PDMS microchannel device was also used as a testing device.

Cells were trypsinized from the flasks and suspended at 10^6^ cells/mL [42]. To assure better cell adhesion, 100 µl gelatin was injected into the inlet reservoir of the microchannel and incubated for 2 h prior to cell culture. After rinsing with PBS, the concentrated cell suspension was injected into the gelatin-coated microchannel device using a syringe. The device was incubated at 37°C with 5% CO_2_ for 1.5 h to allow initial cell attachment and spreading on the channel surfaces. Observations were made after 1.5 h incubation using a Nikon phase-contrast microscope, and images were taken with a CCD camera.

To investigate adhesion strength, attached cells were subjected to increasing levels of flow-induced shear stress over a 12 min period. Cell culture medium was driven through the microchannel by a programmable syringe pump (Harvard Apparatus PhD 2000, USA) at 2.775, 5.55, 11.1, 27.75, 55.5, 111 mL/h, each for 2 min. These flow rates translated to shear stress of 0.25, 0.5, 1, 2.5, 5 and 10 dyn/cm^2^. The shear stress was calculated from Equation (1) [43], [44].
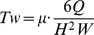
(1)


Where 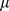 is the viscosity of the flow fluid (0.007 g/cm/s, at 37°C), 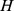 the channel height (0.012 cm), 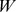 the channel width (0.9 cm) and 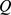 the flow rate (mL/s). Images were taken at 2 min intervals shear period at the same place.

### Statistical analysis

Results are presented as means ± standard error of the mean (SEM). Statistical significance of differences was determined using Student's *t*-test.

## Results

### Cell distribution

**Figure 4:**
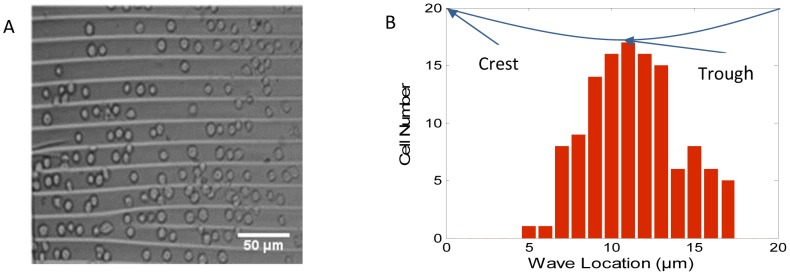
Cell distribution at the initial seeding. (A) Microscope image of cells on a wavy surface with 20 µm spacing and 6.6 µm height; (B) The number of endothelial cells at different wave locations.

BAOECs were seeded onto the wavy surface with a wavelength of 20 µm. At the initial seeding, the rounded BAOECs were almost uniformly distributed on the wavy surface (Fig. 4A). To quantify the cell distribution, the 20 µm wavelength was divided into 1 µm segment locations and a histogram of the number of cells in each segment was created (Fig. 4B). In the phase-contrast microscope image, the white lines represent the crest of the micro-wavy surface. Most cells tend to be located at the troughs of the wavy surface, which demonstrates that wavy surfaces have good control of cell position.

### Cell spreading and alignment

**Figure 5:**
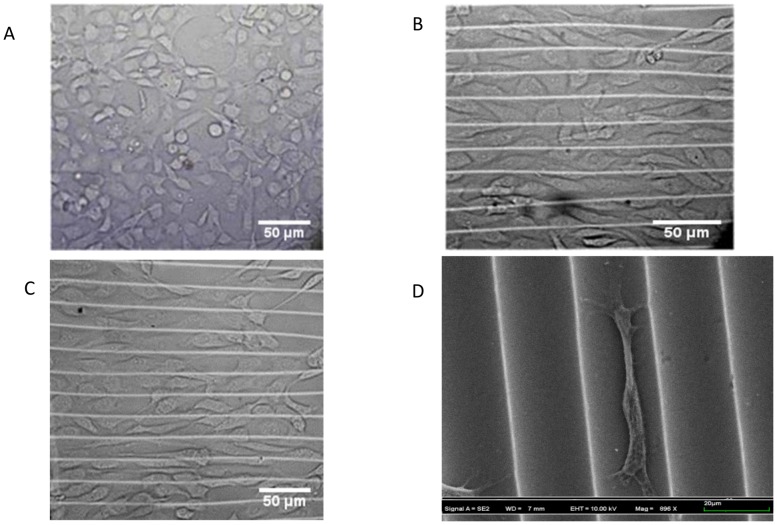
Images of BAOECs on different substrates. (A) Microscope image of cells on flat PDMS substrate after 24 h; (B) Microscope image of cells on 20 µm spacing, 6.6 µm height micro-wave after 24 h; (C) Microscope image of cells on the micro-wave after 48 h; (D) SEM image of a BAOEC on micro-wavy substrate after 24 h. In Fig. D, the cell looks smaller than in Fig. A–C because of the dehydration process in preparation for SEM.

After 24 h of cell culturing, while BAOECs on the flat surface were randomly oriented (Fig. 5A), those on the micro-wavy surface were found oriented along the long axis of the waves (Fig. 5B). The BAOECs on the flat surface also appeared rounder than those on the wavy surface. Comparing the images of the wavy surface captured after 24 h and 48 h (Fig. 5B and 5C), no significant differences were observed on the distribution or spreading of the cells; thus, the incubation time of 24 h was utilized for further analysis. In addition, scanning electron microscopy (SEM) images revealed that the cell aligned along the wave and was located in the trough of the wave (Fig. 5D). In preparation for SEM, cells were fixed in 4% paraformaldehyde for 1 h and washed with PBS; then, the cells were dehydrated in 20%, 30%, 50%, 70%, 85%, 95%, and 100% (volume/volume) ethanol concentration gradient solutions (each for 15 min), followed by 24 h dry out in the refrigerator. Thus, in the SEM image (Fig. 5D), cells look smaller than in the optical images (Fig. 5 A–C).

The cell alignment after static seeding for 24 h was quantified using the Image J software to determine the location and alignment angle of cells. The alignment angle (0°∼90°) of each cell was defined as the angle between the long axis of the cells and the wave direction of the wavy surface. Around 100 cells were measured after 24 h on wavy surface and a statistical analysis was performed on the measured data using Matlab.

**Figure 6:**
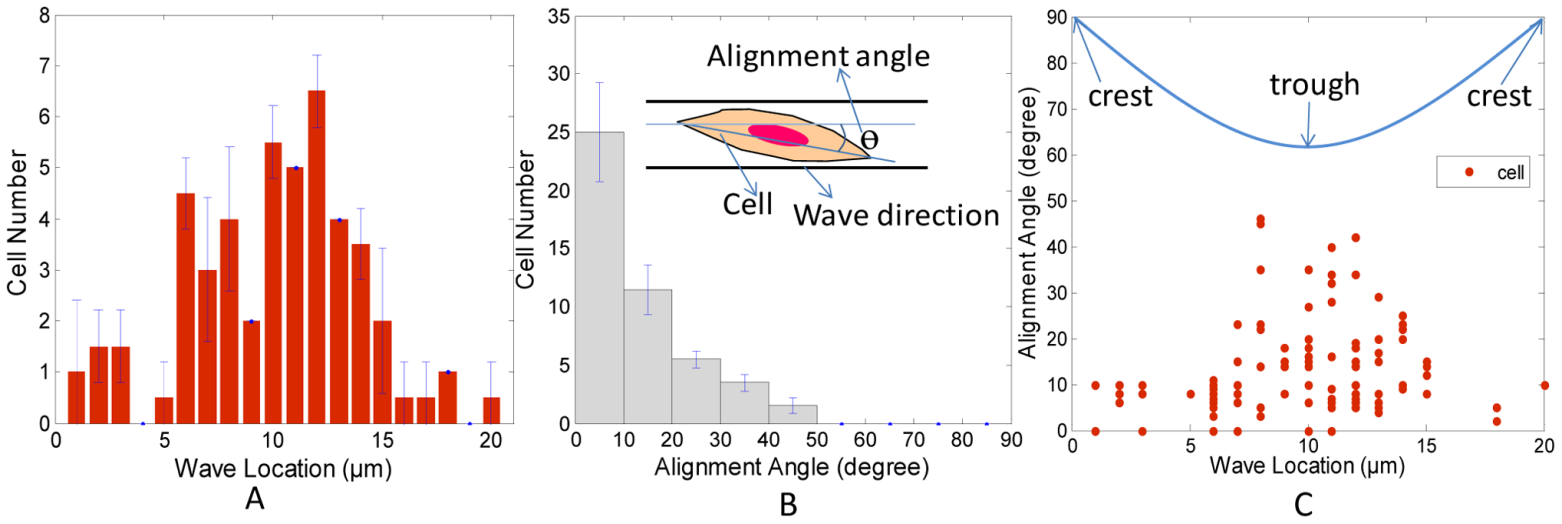
Alignment of BAOECs on 20 µm micro-wavy substrates after 24 h incubation. (A–C) Histograms of cell number, alignment angle, and cell location on 20 µm wavy surfaces (n = 100). Error bars, SEM.

The cell orientation histograms are presented in Fig. 6 A–C. Fig. 6A shows that most cells were located in the troughs of the wave. Fig. 6B shows that about 60% of the cells had a <10° alignment angle, with a significant fraction of them very close to 0° on the 20 µm wavy surface. The spread endothelial cells show strong alignment within the wavy pattern. The alignment angle and wave location of each cell are shown in Fig. 6C. A wider angle distribution of cells is observed at troughs of the wave, while a narrow angle distribution of cells is observed at the crests of the wave.

### Cell spreading on micro-wavy and micro-grooved substrates

**Figure 7:**
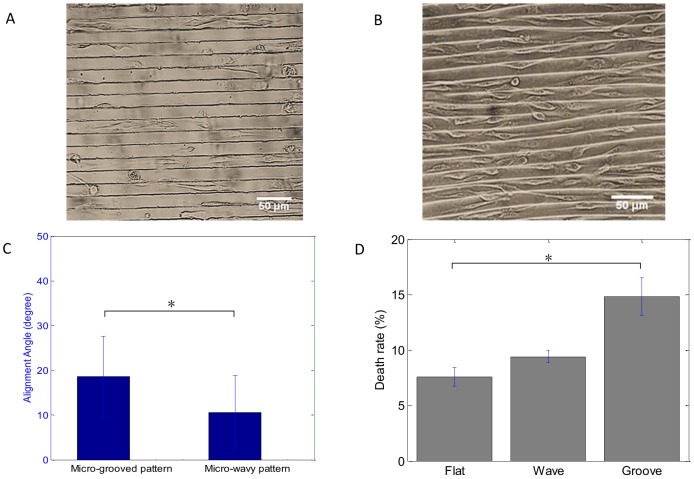
Phase-contrast images of BAOECs after 24 h incubation. (A) 20 µm spacing, 5 µm height micro-groove; (B) 20 µm spacing, 6.6 µm height micro-wave; (C) Alignment angle (mean ± SEM) of BAOECs on micro-grooved and micro-wavy pattern. (D) Death rate of BAOECs on flat, groove and wavy surface. BAOECs on the wavy pattern show better alignment and lower death rate than those on the micro-grooved pattern. *P<0.05; Student's t test.

To understand surface curvature effect to cell adhesion and spreading, we seeded BAOECs onto surfaces with microgrooves for comparison to those on microwaves. Similarly to the micro-wavy pattern, most of the BAOECs were located at the troughs of the grooves after 24 h incubation, as shown in Fig. 7A–B. Fig. 6C shows that cells on the 20 µm wavy surface had better alignment than on the 20 µm groove surface.

Cells were found to not only seed denser on the wavy surface, but also spread healthier, which was quantified by the death rate of cells. Cells on a 20 µm grooved surface had a higher death rate after 24 h than those on the 20 µm wavy pattern and flat surface as depicted in Fig. 7D. This may be explained by the larger area exposed to the culture medium on the wavy surface compared to confinement of the grooved surface.

### Cell adhesion strength

**Figure 8:**
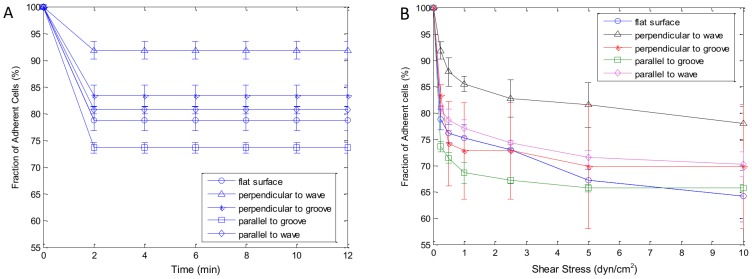
Shear flow testing via a microfluidic based testing platform. (A) Shear stress of 1 dyn/cm^2^ was applied for 12 min; (B) Diagram represents shear stress level applied, 2 min at 0.25 dyn/cm^2^, 2 min at 0.5 dyn/cm^2^, 2 min at 1 dyn/cm^2^, 2 min at 2.5 dyn/cm^2^, 2 min at 5 dyn/cm^2^, 2 min at 10 dyn/cm^2^. Data presented as mean ± SEM (n = 3).

To measure the cell adhesion strength, five control experiments were performed to examine cells detachment over time and as a function of shear stress. The flow was applied in the direction of both perpendicular and parallel to the grooves and waves. In the first experiment, the shear flow was fixed at 1 dyn/cm^2^ for 12 min. This particular shear level corresponds to the lower end of physiological shear stress in the blood vessels which ranges from 1 dyn/cm^2^ to 70 dyn/cm^2^ [45]. The number of attached cells decreased quickly within the first 2 min, and plateaued afterwards, as shown in Fig. 8A. This suggested that at a given shear level cells detached quickly rather than gradually peeled off over time. Thus, two minutes is enough to establish a steady number of cell detachment.

The second experiment was performed by repeating the standard shear assay of 0.25, 0.5, 1, 2.5, 5 and 10 dyn/cm^2^ at 2 min intervals over the 12 min period. We measured percentage of cells stayed in flat, groove and wavy channels at six levels of shear stress (Fig. 8B). In total, three experiments (n = 3) were conducted for each channel with flat, groove and wavy surfaces, respectively. Qualitative evaluation of these profiles showed that BAOECs were attached to flat channels with ∼60% cells remaining after the assay. When the flow direction was perpendicular to the grooves and waves, BAOECs attached better on wavy channel with ∼80% cells remaining after the assay. While when the flow direction was parallel to the grooves and waves, BAOECs on the wavy pattern channels also showed stronger cell adhesion than those on the grooves and flat ones.

## Discussion

In tissue implantation such as vascular graft implantation, cells are often seeded on the surface of scaffolds before implantation. It is important that these scaffolds have the optimal geometry which favors cell attachment, differentiation and proliferation. In particular, this paper analyzed the cell adhesion strength on different substrate geometries which is important for cell seeding on scaffolds as the cells must be able to withstand the local shear stresses in a dynamic in vivo system. In this work, a 20 µm periodic wavy pattern was demonstrated to significantly improve BAOECs orientation and adhesion compared to a flat surface, as shown in Fig. 5 and 8.

Micropatterning has been widely examined for the ability to alter cell morphology, function and its application in tissue engineering. Fu et al. [30] found that when the groove spacing was comparable to the spread cell size, the cells align well in the directions of microgrooves. Their results are consistent with the results obtained from the cell alignment on micro-wavy and micro-grooved PDMS substrates, as shown in Fig. 7 A–C. In this work, stronger alignment effect and lower death rate were observed for cells on the micro-wavy pattern compared to that on a micro-grooved pattern.

Culture of endothelial cells *in vitro* is usually performed under static conditions such as in a culture dish or flask with stationary medium [46]. Since endothelial cells form the inner lining of blood vessels, they are normally exposed to continuously flowing blood as opposed to static conditions. It is therefore desirable to study the cell/biomaterial interactions *in vitro* under conditions closely resembling those *in vivo*, specifically under shear stress after seeding [47]. In the current study, the cells were subjected to shear stress from 0.25 dyn/cm^2^ to 10 dyn/cm^2^ after static spreading on both wavy, groove and flat surface. The results indicated that BAOECs on wavy surface had stronger adhesion strength than cells on flat and groove surface (Fig. 8B). This might be due to the concave shape of the wavy pattern, leading to larger contact area with cell, which is favorable for cell adhesion.

It has been shown that traditional two-dimensional (2D) tissue culture is less physiological than three-dimension (3D) tissue culture [48], [49], [50], [51]. Chen *et al.* described experimental scenarios in which 3D culture is particularly relevant, highlight recent advances in materials engineering for studying cell biology, and discussed examples where studying cells in a 3D context provided insights that would not have been observed in traditional 2D systems [51]. Usually a complex setup using ECM matrix and tracker is needed for such 3D study. Here, the wavy surface with tunable surface topography might allow us to study cell adhesion in a semi-3D environment, which is more cell friendly than a flat and groove surface.

## Conclusions

A micro-wavy pattern has been proposed to influence both the initial seeding and orientation of cells. Most cells are found to stay in troughs of the wavy pattern, which is similar to that on the groove pattern. Meanwhile, BAOECs on the micro-wavy pattern demonstrate lower death rate than that on micro-grooved pattern. More importantly, BAOECs adhered to the wavy surface, displaying both improved orientation and adhesion strength compared to that on the flat surface. Although BAOECs are used in this study, the enhanced alignment and adhesion observed should be applicable to various other cell lines. Our findings suggest that this micro-wavy pattern could be utilized in cell culture for studying cell biology and in cell seeding as a tissue scaffold surface pattern to improve cell adhesion.
